# A Case of Difficult Parturition

**Published:** 1800-01

**Authors:** John Clarke

**Affiliations:** Surgeon, at Alcester, Warwickshire


### *A Cafe of difficult Parturition, by* Mr. Purton, *Surgeon, at Alcefter, Warwickfhire; with Obfervations by* John Clarke, M. D.

---

### *To the Editors of the Medical and Phyfical Journal,*

Gentlemen,

I HAVE lately received the following cafe from Mr. Purton, a very fenfible and judicious furgeon, of Alcefter, in Warwick- fhire; you will, probably, think it deferving of a place in your Journal.

To

To Dr. CLARKE.

DEAR SIR, *Alcefter, Dec.* 3, 1799.

I beg leave to communicate to you a remarkable cafe of difficult parturition, which has fallen under my care lately, and I truft that you will agree with me, that the conduct which I purfued was adapted to the circumftances of the cafe.

Mrs. P. a hard-working woman, about fix and thirty, has been the mother of four children. Her two firft labours were very fevere and lingering, the laft more fo than the firft; as, in the former, the child was born by the natural pains; and, in the latter, with much difficulty, by the forceps. The third labour, fhe had lingering pains for three or four days, and I was with her for moft of the time; at length, the os uteri was fully dilated, and the membranes burft. The head, with the anterior fontanelle towards the left inguen was diftinctly felt at the brim of the pelvis. The pains now very foon increafed in ftrength, and, after a little time, the head advanced fomewhat further; but at laft, although I waited patiently many hours, it did not move in the leaft degree. Confidering the prefentation and the head not being low enough for the application of the forceps, I defired a confultation, and therefore fent over for Mr. Bloxam's affiftance. As foon as he arrived, he thought it proper to turn the child, which was done, and with the greateft difficulty the child was delivered, although I affifted him with all my ftrength; the head was nearly feparated from the body in the attempt, and a confiderable indentation was obfervable on the fide of the head, after the child was born. On examination, there was very confiderable projection of the facrum, fo as to leffen that diameter of the pelvis full an inch and a half. The fourth labour happened about fix weeks ago. The head, in this cafe, did not advance fo far in the pelvis, although the pains were much ftronger, and continued fo for fome time: at length the woman became fo exhaufted, that I thought it prudent to deliver her; but, fortunately, during this deliberation in my mind, the funis was forced down, and it very foon ceafed pulfating. When this took place, I did not hefitate a moment how to proceed; I immediately relieved the mother by evacuating the head, which operation, of courfe, I performed with much lefs violence to my own feelings when I was convinced the child was dead. It was with much difficulty I brought the child into the world, after I had diminifhed the head; and, on examination, the protuberance of the facrum was much increafed fince the laft labour, which fhews that the bones muft have been gradually becoming worfe for many years; and alfo explains why the two firft labours terminated fo favourably.

The

The woman recovered (of which you have had, no doubt, many inftances in fimilar cafes) more rapidly than many do in the beft of times. I am,

Dear Sir,

Your friend and fervant,

T. PURTON.

---

The foregoing cafe appears to me to be worthy of being recorded, becaufe it ferves to fhew the gradual fteps of the difeafe, which is called *mollities offium*, and the manner in which the pelvis, though it may have been once perfect, becomes more and more diftorted, till it is abfolutely incapable of admitting the paffage of a child's head through it, without being diminifhed in fize by evacuating the brain.

The firft labour was completed, as it appears, by the natural efforts. In the fecond, the head was brought through (not, however, without difficulty) by the application of the forceps. In the third, the head did not advance far enough to admit of the application of that inftrument, partly from the face being placed towards the groin, but principally, as is moft likely, from the increafing deformity of the patient's pelvis. The child, in this labour, was turned, and it was delivered by the feet. The head, however, was brought through, not without great force. Between the third and fourth labor, the difeafe continuing to make progrefs, the deformity became ftill greater, fo that it was neceffary to deliver by the crotchet.

I do not know that there is any inftance recorded in which mollities offium, once begun, has been by any means arrefted in its progrefs; the confequence of which is, that the pelvis, firft, for obvious reafons, and afterwards the whole fkeleton, muft give way to the fuperincumbent weight, and if parturition has been rendered difficult from this caufe, the difficulty may be expected to be aggravated in each fucceeding labour.

Not fo in the deformity arifing from rickets. If the unfortunate fubjects of this difeafe attain the age of puberty, the fkeleton does not often yield afterwards to preffure. Hence the difficulty of parturition does not in them become increafed at each fucceeding labour, but the deformity remains where the difeafe left it, through life.

The operation, therefore, of bringing on premature labour; or of turning the child (if the full term of pregnancy has been compleated) is applicable chiefly to cafes of deformity, which are the refult of rickets, where we may in any fucceeding labour expect to find the fame dimenfions of the pelvis as exifted in thofe labours which have gone before. In many cafes of deformed formed pelvis ariſing from the latter cauſe, if the dimenſions of the pelvis are accurately noted by the practitioner, the life of a child may be preſerved by bringing on premature labour, which muſt inevitably be loſt, if the full term of utero-geſtation ſhould be allowed to be compleated.

I need not obſerve, that it is only to the ſlighter caſes of deformity, where ſucceſs can be expected from this practice. The ſubject of premature labour has been ſo ably treated by Dr. Denman, in his excellent Syſtem of Midwifery, that I beg to refer your readers to his work. To the practical directions there laid down, I only think it neceſſary to add, that a ſmall male catheter, curved ſo as to take the ſweep of the vagina and cervix uteri, is a very well adapted inſtrument for puncturing the membranes, becauſe the liquor will be readily drawn off through it; and the chance of labour coming on ſoon will be greater, when the waters are more perfectly evacuated; and much of the ſucceſs muſt neceſſarily depend on the proceſs taking place early; if it ſhould not, the child will either die, or, continuing to grow, the intention of perforating the membranes will be defeated.

I am, Gentlemen,

Yours, &c.

JOHN CLARKE.

*New Burlington-ſtreet, Dec.* 13, 1799.

